# Relationship of Fat Mass Index and Fat Free Mass Index With Body Mass Index and Association With Function, Cognition and Sarcopenia in Pre-Frail Older Adults

**DOI:** 10.3389/fendo.2021.765415

**Published:** 2021-12-24

**Authors:** Reshma Aziz Merchant, Santhosh Seetharaman, Lydia Au, Michael Wai Kit Wong, Beatrix Ling Ling Wong, Li Feng Tan, Matthew Zhixuan Chen, Shu Ee Ng, John Tshon Yit Soong, Richard Jor Yeong Hui, Sing Cheer Kwek, John E. Morley

**Affiliations:** ^1^ Division of Geriatric Medicine, Department of Medicine, National University Hospital, Singapore, Singapore; ^2^ Yong Loo Lin School of Medicine, National University of Singapore, Singapore, Singapore; ^3^ Healthy Ageing Programme, Alexandra Hospital, National University Health System, Singapore, Singapore; ^4^ Department of Geriatrics Medicine, Ng Teng Fong General Hospital, Singapore, Singapore; ^5^ Division of Advanced Internal Medicine, Department of Medicine, National University Hospital, Singapore, Singapore; ^6^ National University Polyclinics, National University Health System, Singapore, Singapore; ^7^ Division of Geriatric Medicine, Saint Louis University School of Medicine, St. Louis, MO, United States

**Keywords:** body composition, fat mass index, fat-free mass index, sarcopenia, physical function, cognition

## Abstract

**Background:**

Body mass index (BMI) is an inadequate marker of obesity, and cannot distinguish between fat mass, fat free mass and distribution of adipose tissue. The purpose of this study was twofold. First, to assess cross-sectional relationship of BMI with fat mass index (FMI), fat free mass index (FFMI) and ratio of fat mass to fat free mass (FM/FFM). Second, to study the association of FMI, FFMI and FM/FFM with physical function including sarcopenia, and cognition in pre-frail older adults.

**Methods:**

Cross-sectional study of 191 pre-frail participants ≥ 65 years, 57.1% females. Data was collected on demographics, cognition [Montreal Cognitive Assessment (MoCA)], function, frailty, calf circumference, handgrip strength (HGS), short physical performance battery (SPPB) and gait speed. Body composition was measured using InBody S10. FMI, FFMI and FM/FFM were classified into tertiles (T1, T2, T3) with T1 classified as lowest and T3 highest tertile respectively and stratified by BMI.

**Results:**

Higher FFMI and lower FM/FFM in the high BMI group were associated with better functional outcomes. Prevalence of low muscle mass was higher in the normal BMI group. FMI and FM/FFM were significantly higher in females and FFMI in males with significant gender differences except for FFMI in ≥ 80 years old. Small calf circumference was significantly less prevalent in the highest tertile of FMI, FM/FMI and FFMI. Prevalence of sarcopenic obesity and low physical function (HGS, gait speed and SPPB scores) were significantly higher in the highest FMI and FM/FFM tertile. Highest FFMI tertile group had higher physical function, higher MoCA scores, lower prevalence of sarcopenic obesity and sarcopenia, After adjustment, highest tertile of FFMI was associated with lower odds of sarcopenia especially in the high BMI group. Highest tertile of FM/FFM was associated with higher odds of sarcopenia. Higher BMI was associated with lower odds of sarcopenia.

**Conclusion:**

FFMI and FM/FFM may be a better predictor of functional outcomes in pre-frail older adults than BMI. Cut-off values for healthy BMI values and role of calf circumference as a screening tool for sarcopenia need to be validated in larger population. Health promotion intervention should focus on FFMI increment.

## Introduction

Population ageing and rise in obesity prevalence worldwide are the two biggest risk factors for non-communicable diseases, including degenerative diseases, sarcopenia, frailty, dementia, increased morbidity, and mortality putting a strain on finite healthcare resources (1–5). Physical inactivity during COVID-19 has further exacerbated the problem (6), and obesity has been recognized as the strongest risk for severe disease and mortality during COVID-19 (7). Elevated body mass index (BMI), waist circumference and/or waist hip ratio are often used to define obesity. BMI need to be interpreted with caution in older adults as loss of physiological height may lead to over-interpretation and lack of correlation with percentage body fat, distribution, or body composition (8–11). Unlike younger adults, high BMI in older adults is associated with better function, cognition, and survival (12–15).

Ageing is known to be associated with body composition changes such as increase in fat mass, decrease in fat free mass and skeletal muscle (8, 16, 17). High BMI is a sum of both fat mass and fat free mass with differing physiological roles better explained by the load-capacity model theory, fat mass (load) and fat free mass (capacity) where the cardio-metabolic diseases, functional disability and sarcopenia risk will depend on the relative contribution of each component to physiological function (18–20). Fat mass distribution and function undergo dramatic changes with ageing where fat mass peaks at 70 years. Thereafter, the fat depot starts to decline with increasing ectopic fat deposition in the epicardium, bone marrow, muscle, liver and other sites leading to loss of lean mass and organ dysfunction (21). Adipose tissue is pro-inflammatory, and obesity is associated with high baseline C-reactive protein, interleukin-6, low adiponectin and high leptin (7). Fat free mass includes body water, skeletal and smooth muscle mass, and bones. Increment of fat free mass has been shown to reduce the negative outcomes of fat mass. Several recent studies have shown that ratio of fat mass to fat free mass (FM/FFM) was associated with cardio-metabolic disorders, non-alcoholic fatty liver disease and adverse outcomes (16, 22, 23). This is thought to be mediated through myokines, osteokines and adipokines regulating muscle, bone, and fat metabolism (24).

Frailty is a multidimensional geriatric syndrome caused by a decline in physiological reserves that increases one’s vulnerability to stressors and associated with increased morbidity and mortality (25). Functional decline associated with frailty is hypothesized to be mediated through pro-inflammatory state due to dysfunctional adipose tissue in obesity and/or redistribution of adipose tissue (26–28). The prevalence of prefrailty in our local population is 37.0% (29). Sarcopenia is a precursor for physical frailty. Currently, there is no gold-standard diagnostic tool for sarcopenia, and it is defined by either reduced muscle strength or physical performance and low appendicular skeletal muscle mass (ASM) (30, 31). There have been many terms used to define loss of muscle mass including “myosteatosis” when muscle mass reduction is accompanied by fat and connective tissue infiltration affecting muscle quality, sarcopenic obesity defined by reduction in muscle mass accompanied by increase in body fat (2) and “osteo-sarcopenia” when reduction of muscle mass is accompanied by reduction in bone density (32).

The impact and association of BMI and body composition especially fat mass, fat free mass and FM/FFM on physical function, sarcopenia and cognition in pre-frail older adults is still unclear. The purpose of this study was twofold. First, to assess cross-sectional relationship of BMI with fat mass index (FMI), fat free mass index (FFMI) and FM/FFM. Second, to study the association of FMI, FFMI and FM/FFM with physical function including sarcopenia, and cognition in pre-frail older adults.

## Materials and Methods

This was a cross-sectional sample consisted of 191 pre-frail older adults recruited from an ongoing study on effect of multidomain intervention at two primary care clinics i.e. Choa Chu Kang and Bukit Batok National University Polyclinic, Singapore (84.4%), Geriatric Clinic at National University Hospital and Alexandra Hospital, Singapore (5.2%) and community setting (10.4%). Participation was entirely voluntary with Choa Chu Kang National University Polyclinic being the control site. Inclusion criteria included participants who could provide consent and follow instructions as certified by primary care physician or study team, ambulant and pre-frail or frail. Exclusion criteria included nursing home residents and those who were either bedridden or wheelchair bound. Only those with BMI of ≥ 18.5 kg/m^2^ and prefrail were included in the analysis as numbers of those with BMI < 18.5 kg/m^2^ and/or frail were less than 10%.

### Demographics and Covariates

Interview questionnaire was administered by trained research staff on demographics, chronic diseases, medications, falls, function, cognition, frailty, sarcopenia, depression, and perceived health. Multimorbidity was defined as ≥ 2 or more self-reported chronic conditions. Three-minute nutritional screening (3-MinNS) tool was used to assess malnutrition risk (33). Katz activity of daily living (ADL) and Lawton’s activity of daily living (IADL) scale were used to evaluate ADL and IADL respectively (34, 35). Frailty was assessed using the FRAIL scale (Fatigue, Resistance, Aerobic, Illness, and Loss of Weight), where pre-frail was defined as 1-2 with a maximum score of 5 (36). SARC-F was used to screen for sarcopenia (lifting and carrying 10 pounds, walking across a room, transferring from bed/chair, climbing a flight of 10 stairs, and frequency of falls in the past 1 year), where the scores range from 0 to 10, and ≥4 is positive for sarcopenia (37). Cognitive status was assessed using the Montreal Cognitive Assessment (MoCA) (38). Physical activity was assessed using the Rapid Assessment of Physical Activity tool (39). Definition of being physically active was based on the World Health Organization recommendations: ≥ 150 minutes of moderate intensity, or ≥ 75 minutes of vigorous intensity aerobic physical activity per week (40). Depression was evaluated using the 15-item Geriatric Depression Scale (GDS), with ≥ 5 to define depression (41). Mental vitality was based on 3 questions from the GDS: 1) Are you basically satisfied with your life? 2) Do you feel that your life is empty? 3) Do you feel full of energy? A score of zero defined high mental vitality while a score between one to three signified low mental vitality (42). Perceived health was evaluated using the EuroQol vertical visual analogue scale (43).

### Calf Circumference, Waist Circumference and Physical Function

Physical performance test included assessment of handgrip strength (HGS), gait speed and Short Physical Performance Battery test (SPPB). Maximum HGS was measured using Jamar hand dynamometer on the dominant arm in the seated position with elbow flexed at 90°. Poor HGS was defined according to the 2019 Asian Working Group for Sarcopenia criteria with cut-offs of 28kg for males and 18kg for female (31). The SPPB included 3 components on balance, gait speed and chair stand and is scored out of 12 points (4 points per-component). Waist circumference was measured midpoint between the last rib and iliac crest. Calf circumference was measured at maximal circumference in seated position with feet on the floor and leg positioned at 90° using non-elastic tape. Low calf circumference was defined based on the 2019 Asian Working Group for Sarcopenia criteria with calf circumference cut-offs of < 34 cm for males and <33 cm for female (31).

### Measurement of BMI and Body Composition

BMI was calculated by dividing body weight (kg) by height squared (m^2^). Normal BMI cut-off was based on WHO recommendations for Asians (44) of 23.0 kg/m^2^ and any value above it was regarded as high BMI. A multi-frequency bioelectrical impedance analyzer, InBody S10 was used to estimate body composition including fat mass, fat free mass, body cell mass, ASM (kg/m^2^) and whole-body phase angle. Inbody bioelectrical impedance analyzer has been validated in different populations including older adults and Asians (45). FMI and FFMI were calculated as fat mass divided by height squared and fat free mass divided by height squared, respectively. FMI, FFMI and FM/FFM were classified into tertiles (T1, T2, T3) with T1 classified as lowest and T3 highest tertile respectively and stratified by BMI. Low muscle mass was defined based on to the 2019 Asian Working Group for Sarcopenia criteria with ASM/height² cut-offs of <7.0 kg/m^2^ for males and <5.7 kg/m^2^ for females (31). Sarcopenia diagnosis was based on the 2019 Asian Working Group for Sarcopenia criteria of gender specific cut offs for ASM/height² and either low HGS based on gender specific cut offs or slow gait speed of <1m/s (31). Sarcopenic obesity was defined as having an ASM of less than 19.75kg and having a percentage body fat of more than 25% for males, and ASM less than 15.02kg and percentage body fat of more than 35% for females (46).

### Statistical Analysis

Our study data was analyzed using IBM SPSS Version 26.0. Categorical variables were presented as frequencies and continuous variables are presented as mean ± Standard Deviation in **Tables 1** and **2**. Chi-squared test with Bonferroni correction was used to determine significance between categorical variables. One-Way ANOVA with Tukey test and Welch ANOVA with Games-Howell were used for continuous variables, with and without homogeneity of variance assumption respectively.

**Table 1:** Demographics, physical function, cognitive function and body composition based on FMI, FFMI and FM/FFM tertiles.

Variables		All n=191 (100.0)	Fat Mass Index (FMI)				Fat-Free Mass Index (FFMI)				Fat Mass/ Fat-Free Mass (FM/FFM)			
			Tertile 1 n=59 (30.9)	Tertile 2 n=66 (34.6)	Tertile 3 n=66 (34.6)	P value	Tertile 1 n=59 (30.9)	Tertile 2 n=66 (34.6)	Tertile 3 n=66 (34.6)	P value	Tertile 1 n=64 (33.5)	Tertile 2 n=66 (34.6)	Tertile 3 n=61 (31.9)	p value
	**Demographics**													
	Age	73 ± 5.7	72.6 ± 6.0	73.4 ± 5.7	73.0 ± 5.7	0.78	73.2 ± 6.0	74.1 ± 6.0	71.8 ± 5.1	0.08	72.5 ± 5.8	74 ± 5.8	72.5 ± 5.5	0.24
	Gender^#^					**<0.01**				**<0.01**				**<0.01**
	Male	82 (100.0)	40 (48.8)^a^	31 (37.8)^b^	11 (13.4)^a,b^		9 (11.0)^a,b^	27 (32.9)^a,c^	46 (56.1)^b,c^		50 (61.0)^a,b^	24 (29.3)^a,c^	8 (9.8)^b,c^	
	Female	109 (100.0)	19 (17.4)^a^	35 (32.1)^b^	55 (50.5)^a,b^		50 (45.9)^a,b^	39 (35.8)^a,c^	20 (18.3)^b,c^		14 (12.8)^a,b^	42 (38.5)^a,c^	53 (48.6)^b,c^	
	Ethnicity^#^					0.53				0.51				0.40
	Chinese	158 (100.0)	51 (32.3)	55 (34.8)	52 (32.9)		50 (31.6)	55 (34.8)	53 (33.5)		54 (34.2)	57 (36.1)	47 (29.7)	
	Malay	13 (100.0)	2 (15.4)	4 (30.8)	7 (53.8)		4 (30.8)	2 (15.4)	7 (53.8)		4 (30.8)	2 (15.4)	7 (53.8)	
	Indian	19 (100.0)	5 (26.3)	7 (36.8)	7 (36.8)		5 (26.3)	8 (42.1)	6 (31.6)		5 (26.3)	7 (36.8)	7 (36.8)	
	BMI (kg/m^2^)	25.9 ± 4.2	22.4 ± 2.8^a,b^	25.2 ± 2.4^a,c^	29.7 ± 3.5^b,c^	**<0.01**	23.2 ± 2.9^a,b^	26 ± 3.4^a,c^	28.3 ± 4.4^b,c^	**<0.01**	23.5 ± 3.4^a^	24.7 ± 2.7^b^	29.7 ± 3.6^a,b^	**<0.01**
	BMI status					**<0.01**				**<0.01**				**<0.01**
	Normal BMI	50 (26.2)	38 (64.4)^a,b^	11 (16.7)^a,c^	1 (1.5)^b,c^		32 (54.2)^a,b^	13 (19.7)^a^	5 (7.6)^b^		32 (50.0)^a,b^	17 (25.8)^a,c^	1 (1.6)^b,c^	
	High BMI	141 (73.8)	21 (35.6)	55 (83.3)	65 (98.5)		27 (45.8)	53 (80.3)	61 (92.4)		32 (50.0)^a,b^	49 (74.2)^a,c^	60 (98.4)^b,c^	
	Education (years)	7.8 ± 4.3	8.5 ± 4.5	8.1 ± 3.6	6.9 ± 4.5	0.10	7.1 ± 4.7	7.8 ± 4.2	8.4 ± 4	0.26	8.9 ± 4.2^a^	7.8 ± 3.8	6.6 ± 4.6^a^	**0.01**
	Small calf circumference	38 (21.0)	22 (37.9)^a^	13 (21.0)^b^	3 (4.9)^a,b^	**<0.01**	22 (40.0)^a,b^	12 (19.0)^a^	4 (6.3)^b^	**<0.01**	18 (29.0)^a^	18 (28.6)^b^	2 (3.6)^a,b^	**<0.01**
	Waist circumference* (cm)	93.1 ± 11.0	85.2 ± 6.2^a,b^	92.6 ± 9.0^a,c^	100.1 ± 11.6^b,c^	**<0.01**	86.9 ± 8.3^a,b^	94.9 ± 11.9^a,c^	97.2 ± 9.8^b,c^	**<0.01**	88.8 ± 6.9^a^	91.1 ± 10.8^b^	100.2 ± 11.4^a,b^	**<0.01**
	Depression	59 (31.6)	15 (25.4)	20 (31.7)	24 (36.9)	0.39	23 (41.1)	22 (33.8)	14 (21.2)	0.06	15 (23.4)	22 (34.4)	22 (37.3)	0.21
	Diabetes	98 (51.3)	25 (42.4)^a^	31 (47.0)	42 (63.6)^a^	**0.04**	20 (33.9)^a,b^	37 (56.1)^a^	41 (62.1)^b^	**<0.01**	30 (46.9)	28 (42.4)^a^	40 (65.6)^a^	**0.02**
	Hyperlipidaemia	157 (82.2)	48 (81.4)	52 (78.8)	57 (86.4)	0.51	42 (71.2)^a^	56 (84.8)	59 (89.4)^a^	**0.02**	54 (84.4)	50 (75.8)	53 (86.9)	0.22
	Hypertension	138 (72.6)	41 (69.5)	51 (77.3)	46 (70.8)	0.57	38 (65.5)	49 (74.2)	51 (77.3)	0.32	47 (73.4)	48 (72.7)	43 (71.7)	0.98
	Multimorbidity (≥ 2 or more conditions)	160 (83.8)	48 (81.4)	54 (81.8)	58 (87.9)	0.53	45 (76.3)	58 (87.9)	57 (86.4)	0.17	52 (81.3)	54 (81.8)	54 (88.5)	0.47
	Low mental vitality	107 (57.2)	24 (40.7)^a^	38 (60.3)	45 (69.2)^a^	**<0.01**	37 (66.1)	37 (56.9)	33 (50.0)	0.20	31 (48.4)	36 (56.3)	40 (67.8)	0.09
	Nutritional status (MNA)	21.8 ± 2.1	21.4 ± 2.5^a^	22.4 ± 1.8^a^	21.7 ± 2.0	**0.04**	21.4 ± 2.1	22.1 ± 2.0	22.0 ± 2.2	0.19	21.4 ± 2.6	22.3 ± 1.6	21.7 ± 2.0	0.05
	≥ 5% weight loss in past year	19 (9.9)	10 (16.9)	3 (4.5)	6 (9.1)	0.07	4 (6.8)	6 (9.1)	9 (13.6)	0.42	10 (15.6)	4 (6.1)	5 (8.2)	0.16
	At least 1 fall in past year	50 (26.2)	13 (22.0)	16 (24.2)	21 (31.8)	0.42	14 (23.7)	20 (30.3)	16 (24.2)	0.64	13 (20.3)	19 (28.8)	18 (29.5)	0.42
	At least one ADL impairment	32 (16.8)	4 (6.8)	15 (22.7)	13 (19.7)	**0.04**	10 (16.9)	11 (16.7)	11 (16.7)	1.00	8 (12.5)	13 (19.7)	11 (18.0)	0.52
	At least one IADL impairment	56 (29.3)	17 (28.8)	20 (30.3)	19 (28.8)	0.98	21 (35.6)	18 (27.3)	17 (25.8)	0.44	21 (32.8)	18 (27.3)	17 (27.9)	0.75
	Perceived Health (EQ-VAS)	69.4 ± 14.8	70.6 ± 16.2	70.7 ± 13.8	67 ± 14.4	0.28	67.3 ± 16.0	69.5 ± 15.4	71 ± 12.9	0.37	72.5 ± 13.9	68.3 ± 15.9	67.2 ± 14.2	0.10
	Physically active	30 (15.8)	14 (24.1)	9 (13.6)	7 (10.6)	0.10	6 (10.2)	8 (12.3)	16 (24.2)	0.06	14 (22.2)	9 (13.6)	7 (11.5)	0.22
	Physical activity (Rapid Assessment of Physical Activity)	3.4 ± 1.6	3.9 ± 1.6^a,b^	3.0 ± 1.6^a^	3.2 ± 1.4^b^	**<0.01**	3.0 ± 1.4^a^	3.1 ± 1.6^b^	3.9 ± 1.7^a,b^	**<0.01**	3.9 ± 1.6^a^	3.0 ± 1.6^a^	3.2 ± 1.5	**<0.01**
	Physical and cognitive function													
	Sarcopenia (SARC-F)	1.6 ± 1.8	0.8 ± 1.4^a,b^	1.5 ± 1.7^a^	2.3 ± 2.0^b^	**<0.01**	1.8 ± 2.0	1.8 ± 1.9	1.2 ± 1.5	0.05	0.8 ± 1.3^a,b^	1.6 ± 1.7^a,c^	2.4 ± 2.0^b,c^	**<0.01**
	Sarcopenia (AWGS 2019)	45 (23.7)	18 (30.5)	14 (21.2)	13 (20.0)	0.33	32 (55.2)^a,b^	10 (15.2)^a^	3 (4.5)^b^	**<0.01**	13 (20.3)	19 (28.8)	13 (21.7)	0.48
	Sarcopenic obesity	59 (30.9)	10 (16.9)^a^	15 (22.7)^b^	34 (51.5)^a,b^	**<0.01**	27 (45.8)^a^	23 (34.8)^b^	9 (13.6)^a,b^	**<0.01**	10 (15.6)^a^	15 (22.7)^b^	34 (55.7)^a,b^	**<0.01**
	Handgrip strength, mean (kg)	22.2 ± 7.2	24.9 ± 6.7^a^	22.7 ± 7.8^b^	19.2 ± 5.8^a,b^	**<0.01**	18.4 ± 5.1^a,b^	21.4 ± 6.5^a,c^	26.4 ± 7.3^b,c^	**<0.01**	26.2 ± 6.7^a,b^	21.6 ± 7.0^a,c^	18.6 ± 5.7^b,c^	**<0.01**
	Gait speed (m/s)	0.9 ± 0.3	1.0 ± 0.3^a,b^	0.8 ± 0.2^a^	0.8 ± 0.2^b^	**<0.01**	0.9 ± 0.3	0.9 ± 0.2	0.9 ± 0.3	0.20	1.0 ± 0.3^a,b^	0.8 ± 0.3^a^	0.8 ± 0.2^b^	**<0.01**
	SPPB score	9.6 ± 2.3	10.3 ± 1.8^a,b^	9.3 ± 2.6^a^	9.3 ± 2.3^b^	**<0.01**	9.2 ± 2.4^a^	9.4 ± 2.4	10.3 ± 2.0^a^	**0.02**	10.3 ± 1.8^a,b^	9.3 ± 2.6^a^	9.2 ± 2.4^b^	**<0.01**
	Handgrip strength (kg)	22.2 ± 7.2	24.9 ± 6.7^a^	22.7 ± 7.8^b^	19.2 ± 5.8^a,b^	**<0.01**	18.4 ± 5.1^a,b^	21.4 ± 6.5^a,c^	26.4 ± 7.3^b,c^	**<0.01**	26.2 ± 6.7^a,b^	21.6 ± 7.0^a,c^	18.6 ± 5.7^b,c^	**<0.01**
	MoCA	24.9 ± 4.3	25.1 ± 4.7	25.3 ± 3.7	24.4 ± 4.4	0.48	23.6 ± 5.3a	25.2 ± 3.9	25.8 ± 3.2a	**0.03**	25.7 ± 3.0	24.9 ± 5.0	24.2 ± 4.5	0.10
	**Body composition**													
	Appendicular Skeletal muscle Mass (kg/m^2^)	7.1 ± 1.7	6.8 ± 1.1	7.4 ± 2.1	7.0 ± 1.7	0.15	5.9 ± 1.1^a,b^	7.1 ± 1.3^a,c^	8.2 ± 1.7^b,c^	**<0.01**	Higher pre	7.1 ± 2.0	6.7 ± 1.2^a^	**0.02**
	Low muscle mass	57 (29.8)	24 (40.7)	19 (28.8)	14 (21.2)	0.06	40 (67.8)^a,b^	14 (21.2)^a,c^	3 (4.5)^b,c^	**<0.01**	18 (28.1)	25 (37.9)	14 (23.0)	0.17
	Whole Body Phase Angle (50Khz), mean	4.9 ± 0.9	5.2 ± 0.9^a^	4.9 ± 1.0	4.8 ± 0.8^a^	**<0.01**	4.6 ± 0.8^a^	4.9 ± 0.8^b^	5.3 ± 1.0^a,b^	**<0.01**	5.3 ± 1.0^a,b^	4.8 ± 0.9^a^	4.8 ± 0.7^b^	**<0.01**
	Obese (by fat mass percentage)	119 (62.3)	12 (20.3)^a,b^	41 (62.1)^a,c^	66 (100.0)^b,c^	**<0.01**	31 (52.5)	44 (66.7)	44 (66.7)	0.18	21 (32.8)^a,b^	37 (56.1)^a,c^	61 (100.0)^b,c^	**<0.01**
	Body fat	21.4 ± 7.3	13.9 ± 2.3^a,b^	20.2 ± 2.7^a,c^	29.4 ± 5.2^b,c^	**<0.01**	19.1 ± 5.9^a,b^	22.3 ± 7.1^a^	22.7 ± 8.3^b^	**<0.01**	14.9 ± 3.4^a,b^	20.2 ± 3.6^a,c^	29.7 ± 5.2^b,c^	**<0.01**
	Body fat percentage	33.5 ± 8.7	24.2 ± 4.3^a,b^	32.2 ± 2.9^a,c^	43.1 ± 4.7^b,c^	**<0.01**	35.2 ± 8.1^a^	34.4 ± 8.0	31.1 ± 9.5^a^	**0.02**	24.1 ± 3.9^a,b^	33.1 ± 2.1^a,c^	43.7 ± 4.3^b,c^	**<0.01**
	Fat Free Mass	42.1 ± 8.2	44.5 ± 8.5^a^	43.1 ± 8.6^b^	38.9 ± 6.6^a,b^	**<0.01**	34.6 ± 4.3^a,b^	41.4 ± 4.6^a,c^	49.5 ± 7.3^b,c^	**<0.01**	47.1 ± 8.0^a,b^	40.9 ± 7.9^a^	38.2 ± 6.1^b^	**<0.01**
	Visceral fat area (cm^2^)	100.1 ± 46.1	60.9 ± 11.6^a,b^	86.8 ± 25.1^a,c^	148.4 ± 38.9^b,c^	**<0.01**	96.3 ± 42.3	103.8 ± 46.7	99.8 ± 48.9	0.67	62.5 ± 14.9^a,b^	85.9 ± 27.1^a,c^	155 ± 30.0^b,c^	**<0.01**
	Body cell mass	25.7 ± 7.2	28.8 ± 5.7^a,b^	25.3 ± 8.6^a^	23.3 ± 6.0^b^	**<0.01**	21.4 ± 4.5^a,b^	25.1 ± 6.1^a,c^	30.1 ± 7.8^b,c^	**<0.01**	29.6 ± 6.3^a,b^	23.6 ± 8.3^a^	23.8 ± 5.1^b^	**<0.01**

Bold indicates significance; Values are n (%), otherwise mean ± Standard Deviation (SD); Each superscript denotes significant difference from each other at p<0.05; ^#^Row %, otherwise column %; *n=111; BMI, Body Mass Index; ADL, Activities of Daily Living; IADL, Instrumental Activities of Daily Living; EQ-VAS, EuroQol Visual Analogue Scale; RAPA, Rapid Assessment of Physical Activity; AWGS, Asian Working Group for Sarcopenia; MNA, Mini Nutritional Assessment; SPPB, Short Physical Performance Battery; MoCA, Montreal Cognitive Assessment.

**Table 2:** Body composition by gender and BMI stratification.

	Male n=82 (42.9)		Female n=109 (57.1)		p value
	Normal BMI n=24 (12.6)	High BMI n=58 (30.4)	Normal BMI n=26 (13.6)	High BMI n=83 (43.5)	
Appendicular Skeletal muscle Mass (kg/m2), mean	6.8 ± 0.8^a,b^	8.2 ± 1.9^a,c,d^	5.5 ± 0.6^b,c,e^	6.9 ± 1.5^d,e^	**<0.01**
Low muscle mass	13 (54.2)^a,b^	13 (22.4)^a,c^	17 (65.4)^c,d^	14 (16.9)^b,d^	**<0.01**
Whole Body Phase Angle (50Khz), mean	5.1 ± 0.8	5.1 ± 1.1	4.6 ± 0.8	4.9 ± 0.8	0.06
Obese (by fat mass %)	7 (29.2)^a,b^	46 (79.3)^a,c^	3 (11.5)^c,d^	63 (75.9)^b,d^	**<0.01**
Fat mass, mean	12.9 ± 2.7^a,b,c^	21.6 ± 5.8^a,d,e^	15.7 ± 2.9^b,d,f^	25.6 ± 6.8^c,e,f^	**<0.01**
Fat mass (%), mean	22.5 ± 4.9^a,b,c^	30.2 ± 7.1^a,d^	31.0 ± 4.6^b,e^	39.7 ± 6.7^c,d,e^	**<0.01**
Fat Free Mass, mean	45.0 ± 6.9^a,b,c^	49.6 ± 7.2^a,d,e^	34.8 ± 3.6^b,d,f^	38.3 ± 5.4^c,e,f^	**<0.01**
Visceral fat area (cm^2^)	58.7 ± 14.6^a,b,c^	91.5 ± 36.5^a,d^	76.2 ± 22.9^b,e^	125.6 ± 48.7^c,d,e^	**<0.01**
Body cell mass, mean	29.1 ± 4.6^a,b^	30.0 ± 8.0^c,d^	22.3 ± 2.5^a,c^	22.8 ± 6.4^b,d^	**<0.01**

Bold indicates significance; Values are mean ± Standard Deviation (SD), otherwise n (%); Each superscript denotes significant difference from each other at p<0.05.

**Figure 1:**
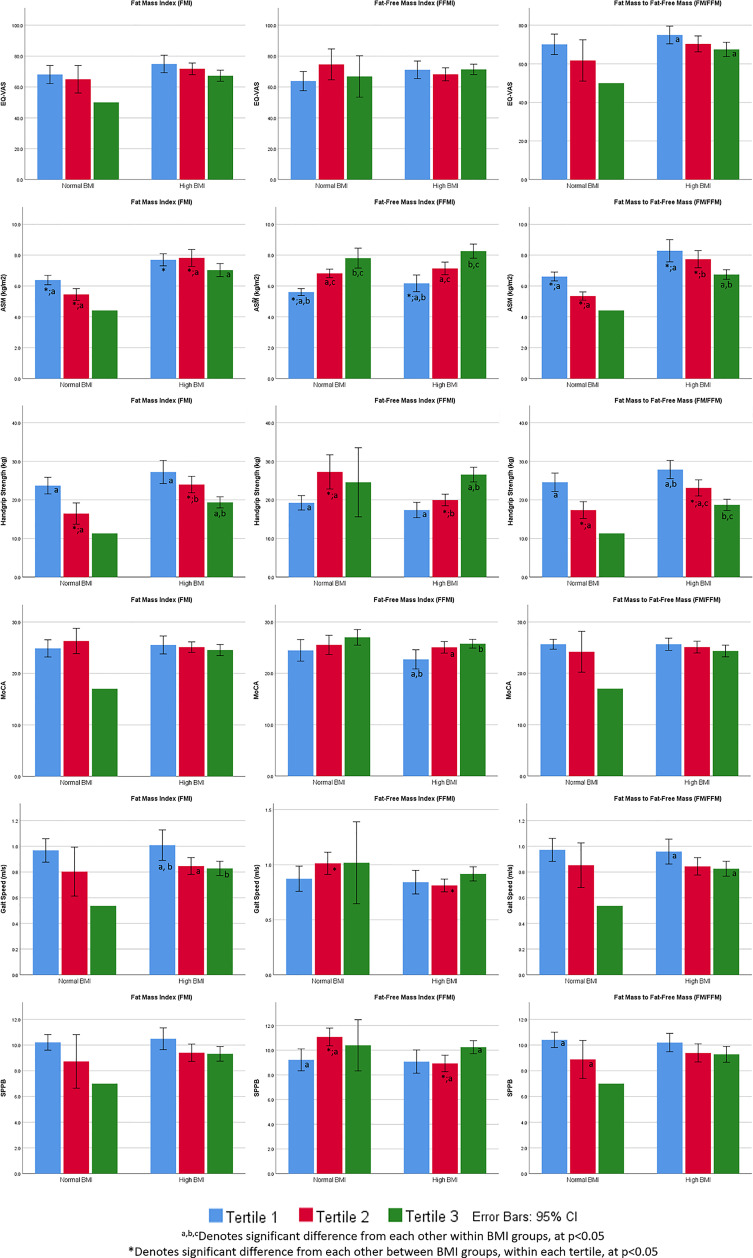
Perceived health, appendicular skeletal muscle mass (kg/m^2^), physical function and cognition by BMI stratification, FMI, FFMI and FM/FFM tertiles.

**Figure 2:**
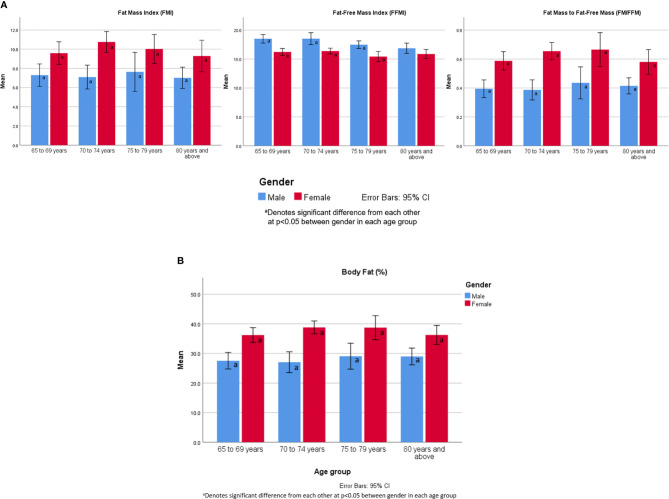
**(A)** FMI, FFMI and FM/FFM values by age group and gender. **(B)** Body fat percentage by age group and gender.

Unadjusted and adjusted logistic and multiple linear regression models were used for predicting sarcopenia and BMI respectively. Sub-group analyses were carried out between BMI groups and gender, for sarcopenia and BMI prediction respectively. FMI, FFMI and FM/FFM were used as predictor variables (in tertiles for logistic regression and continuous in linear regression), with an addition of BMI for logistic regression. Adjustments were made for age, gender and physical activity for logistic regression, and age and physical activity for multiple linear regression. Odds ratios and B-coefficients were obtained as test statistics for logistic and multiple linear regression respectively, with 95% confidence intervals for all models. One-Way ANOVA with Tukey test and Welch ANOVA with Games-Howell were also used in **Figure 1** to test for significant differences within each BMI group (between tertiles). Independent t-test was used for pairwise comparison within each FMI, FFMI and FM/FFM tertile, between BMI groups. Likewise, independent t-test was used to compare differences between gender per age group in **Figures 2A, B**. Statistical significance was set as p<0.05.

### Ethics Approval and Informed Consent

The studies involving human participants were reviewed and approved by The National Healthcare Group Domain Specific Review Board to be conducted at the Choa Chu Kang and Bukit Batok National University Polyclinic, Geriatric Clinic at National University Hospital and Alexandra Hospital and community setting in Singapore. The patients/participants provided their written informed consent to participate in this study.

## Results

The sample comprised of 82 men and 109 women aged ≥ 65 years with a mean age of 73.0 ± 5.7 years and 57.1% were females. They were all classified as pre-frail based on the FRAIL scale. Demographics, clinical characteristics, functional measures, cognitive scores, perceived health, and body composition of the study population stratified according to FMI, FFMI and FM/FMI tertiles are shown in **Table 1**. Those in the lowest FM/FFM tertile had significantly higher education level of 8.9 ± 4.2 years compared with 6.6 ± 4.6 years for highest FM/FFM tertile. Almost one in two females were categorized under the highest FMI and FM/FFM tertile respectively compared with one in eight males for FMI and one in ten males for FM/FMI highest tertile. Mean BMI was significantly higher in the highest tertiles with 29.7 ± 3.5kg/m^2^ for FMI, 28.3 ± 4.4kg/m^2^ for FFMI and 29.7 ± 3.6kg/m^2^ for FM/FMI respectively. The prevalence of small calf circumference was significantly higher in the lowest tertile compared with highest tertile for FMI (37.9% *vs* 4.9%), FFMI (40.0% *vs* 6.3%) and FM/FFM (29.0% *vs* 3.6%). Diabetes prevalence was significantly higher in all the highest tertile compared with lowest tertile for FMI, FFMI and FM/FFM, 63.6% *vs* 42.4%, 62.1% *vs* 33.9% and 65.6% *vs* 46.9% respectively. There was significantly higher prevalence of at least 1 ADL impairment in the higher FMI tertiles, 22.7% in FMI T3, 19.7% in FMI T2 compared with 6.8% in FMI T1. Physical activity levels were significantly higher in the lowest FMI and FM/FFM tertile and highest FFMI tertile.

### Physical and Cognitive Function

Amongst the lowest FFMI tertile group, 55.2% were classified as sarcopenic compared with 4.5% in the highest tertile group. There were no significant differences between tertiles in the prevalence of sarcopenia for FMI and FM/FFM. Prevalence of sarcopenic obesity was highest in the highest tertile compared with lowest tertile for FMI (51.5% *vs* 16.9%) and FM/FFM (55.7% *vs* 15.6%). On the contrary, 45.8% of the lowest FFMI tertile compared with 13.6% of the highest tertile were classified as sarcopenic obesity. SARC-F scores were 3-fold higher in the highest tertile compared with lowest tertile for FMI (2.3 ± 2.0 *vs* 0.8 ± 1.4) and FM/FFM (2.4 ± 2.0 *vs*. 0.8 ± 1.3).

HGS was significantly higher in the lowest tertile compared with highest tertile for FMI (24.9kg ± 6.7 *vs* 19.2kg ± 5.8) and FM/FMI (26.2kg ± 6.7 *vs* 18.6kg ± 5.7). For FFMI, HGS was significantly higher in the highest tertile compared with lowest tertile, 26.4kg ± 7.3 *vs* 18.4kg ± 5.1 respectively. While there was no significant difference in mean gait speed amongst FFMI tertiles, it was significantly lower for FMI T2 and T3 compared with T1 (0.8m/s ± 0.2, 0.8m/s ± 0.2 and 1.0 m/s ± 0.3 respectively) and FM/FFM T2 and T3 compared with T1 (0.8 m/s ± 0.3, 0.8 m/s ± 0.2 and 1.0 m/s ± 0.3 respectively). SPPB results were significantly higher in the FMI T1 (10.3 ± 1.8) compared with T2 (9.3 ± 2.6) and T3 (9.3 ± 2.3). For FM/FFM, SPPB scores were also significantly higher in the T1(10.3 ± 1.8) compared with T2 (9.3 ± 2.6) and T3 (9.2 ± 2.4). For FFMI, significant difference in SPPB scores were only observed between T1 (9.2 ± 2.4) and T3 (10.3 ± 2.0). Mean MoCA scores were significantly higher in the highest FFMI tertile compared with lowest, 25.8 ± 3.2 and 23.6 ± 5.3 respectively.

### Body Composition

There were significant differences in the body composition across the groups. ASM was significantly higher in the highest FFMI tertile T3 (8.2 ± 1.7kg/m^2^) compared to T1 (5.9 ± 1.1kg/m^2^) and T2 (7.1 ± 1.3kg/m^2^). The reverse was true for FM/FFM, where ASM was significantly higher in T1 (7.4 ± 1.7 kg/m^2^) compared with T3 (6.7 ± 1.2kg/m^2^). There was significantly higher prevalence of low muscle mass in the lowest FFMI tertile (67.8%) compared with highest FFMI tertile (4.5%). Whole body phase angle was significantly higher in the lowest compared with the highest FMI tertile (5.2 ± 0.9 *vs* 4.8 ± 0.8) and FM/FMI tertile (5.3 ± 1.0 *vs* 4.8 ± 0.7. Body cell mass was significantly higher in the lowest FMI and FM/FFM tertile, and highest in the highest FFMI tertile.

The prevalence of low muscle amongst those with normal BMI was 65.4% for females and 54.2% for males (**Table 2**). Amongst those with high BMI, 79.3% of males and 75.9% of females were classified as obese by fat mass percentage. Visceral fat area was significantly higher in females with high BMI, 125.6 ± 48.7 cm^2^. Body cell mass was significantly higher in males with normal and high BMI than females across both BMI groups.

### FMI, FFMI and FM/FFM Tertiles by BMI Stratification, Perceived Health, Physical and Cognitive Function

In the high BMI group, lowest FM/FFM tertile group had significantly better perceived health, gait speed, and HGS (**Figure 1**). Higher FFMI tertile group had significantly higher MoCA scores, HGS and SPPB scores. Lowest FMI tertile group had significantly higher gait speed and HGS in the high BMI group. In the normal BMI group, HGS and SPPB scores were significantly higher in FFMI T2 (compared with T1) and lower in FM/FFM T2 (compared with T1).

### FMI, FFMI, FM/FFM and Body Fat Percentage by Age Group and Gender

There were significant differences between gender across all age groups for FMI, FFMI, FM/FFM and body fat percentage except for FFMI ≥ 80 years old (**Figures 2A, B**). There were no significant differences within gender.

### FMI, FFMI, and FFM/FFM Tertiles, BMI Stratification and Association With Sarcopenia

The models were adjusted for age, gender, and physical activity and lowest tertile (T1) was used as reference (**Table 3**). Within the normal BMI group, the odds of being classified as sarcopenic was significant for FM/FFM T2 (OR 7.97, 95% CI 1.28 - 49.74, p = 0.03) and FFMI T2 (OR 0.14, 95% CI 0.02 - 0.94, p = 0.04). In the high BMI group, the odds of being classified as sarcopenic was significant for FM/FFM T3 (OR 7.03, 95% CI 1.16 - 42.62, p = 0.03), FFMI T2 (OR 0.03, 95% CI 0.00 - 0.24, p < 0.01) and FFMI T3 (OR 0.004, 95% CI 0.000 - 0.053, p < 0.01). The odds of being diagnosed with sarcopenia was significant for BMI (OR 0.79, 95% CI 0.71 - 0.89, p<0.01).

**Table 3:** Unadjusted and adjusted regression models of FMI, FFMI and FM/FFM tertiles by BMI categories and association with sarcopenia.

Predictor variables	BMI status	Model type	Tertile 1	Tertile 2 OR (95% CI) p value	Tertile 3 OR (95% CI) p value
Fat Mass Index (FMI)	Normal BMI	Unadjusted	Reference	2.41 (0.60-9.63) p=0.22	–
		Adjusted	Reference	2.18 (0.45-10.59) p=0.34	–
	High BMI	Unadjusted	Reference	1.39 (0.26-7.28) p=0.70	2.19 (0.45-10.71) p=0.33
		Adjusted	Reference	1.47 (0.26-8.53) p=0.67	2.86 (0.48-17.08) p=0.25
Fat-Free Mass Index (FFMI)	Normal BMI	Unadjusted	Reference	0.27 (0.07-1.06) p=0.06	–
		Adjusted	Reference	**0.14 (0.02-0.94)** **p=0.04**	–
	High BMI	Unadjusted	Reference	**0.15 (0.05-0.47)** **p<0.01**	**0.06 (0.02-0.24)** **p<0.01**
		Adjusted	Reference	**0.03 (0.00-0.24)** **p<0.01**	**0.004 (0.000-0.053)** **p<0.01**
Fat Mass/Fat-Free Mass (FM/FFM)	Normal BMI	Unadjusted	Reference	**4.58 (1.28-16.36)** **p=0.02**	–
		Adjusted	Reference	**7.97 (1.28-49.74)** **p=0.03**	–
	High BMI	Unadjusted	Reference	2.50 (0.49-12.89) p=0.27	3.83 (0.80-18.33) p=0.09
		Adjusted	Reference	3.11 (0.55-17.59) p=0.20	**7.03 (1.16-42.62)** **p=0.03**
BMI (continuous)		Unadjusted	**0.79 (0.71-0.89)** **p<0.01**		
		Adjusted	**0.79 (0.71-0.89)** **p<0.01**		

Bold indicates significance; Adjusted for age, gender and physical activity; 95% CI, 95% Confidence Interval; OR, Odds Ratio; BMI, Body Mass Index.

### FMI, FFMI and FM/FFM, Gender and Association With BMI

The models were adjusted for age and physical activity (**Table 4**). The results were significant for FMI, FFMI and FM/FFM in both genders, for both unadjusted and adjusted models. Amongst males, FMI, FFMI and FM/FFM significantly correlated with BMI (β 1.10, 95% CI 0.92 - 1.28, p<0.01; β 1.5, 95% CI 1.13 - 1.86, p<0.01 and β 17.25, 95% CI 13.08 - 21.41, p<0.01 respectively). For females, similarly there was significant correlation of FMI, FFMI and FM/FFM with BMI (β 1.16, 95% CI 1.07 - 1.26, p<0.01; β 1.68, 95% CI 1.32 - 2.04, p<0.01 and β 15.47, 95% CI 12.61 - 18.33, p<0.01 respectively).

**Table 4:** Unadjusted and adjusted regression models of FMI, FFMI and FM/FFM by gender and association with BMI.

Predictor variables	Gender	Model type	R^2^	Standard Error	B-coefficient (95% CI) p value
Fat Mass Index (FMI)	Male	Unadjusted	0.64	2.53	**1.10 (0.91-1.28)** **p<0.01**
		Adjusted	0.67	2.44	**1.10 (0.92-1.28)** **p<0.01**
	Female	Unadjusted	0.85	1.62	**1.16 (1.06-1.25)** **p<0.01**
		Adjusted	0.85	1.64	**1.16 (1.07-1.26)** **p<0.01**
Fat-Free Mass Index (FFMI)	Male	Unadjusted	0.47	3.07	**1.46 (1.11-1.80)** **p<0.01**
		Adjusted	0.48	3.07	**1.50 (1.13-1.86)** **p<0.01**
	Female	Unadjusted	0.44	3.14	**1.64 (1.28-2.00)** **p<0.01**
		Adjusted	0.45	3.13	**1.68 (1.32-2.04)** **p<0.01**
Fat Mass/Fat-Free Mass (FM/FFM)	Male	Unadjusted	0.43	3.17	**16.67 (12.39-20.95)** **p<0.01**
		Adjusted	0.49	3.05	**17.25 (13.08-21.41)** **p<0.01**
	Female	Unadjusted	0.52	2.89	**15.31 (12.51-18.11)** **p<0.01**
		Adjusted	0.53	2.92	**15.47 (12.61-18.33)** **p<0.01**

Bold indicates significance; Adjusted for age and physical activity; 95% CI, 95% Confidence Interval.

## Discussion

Our study has shown that body composition especially higher FFMI and lower FM/FFM were associated with better functional outcomes in the pre-frail. The differences in outcomes were more significant in the higher BMI group. Those with high BMI and in the highest FFMI tertile had much lower odds of being classified as sarcopenic. On the contrary, amongst those with high BMI, highest FM/FFM tertile was associated with higher odds of sarcopenia. There was a higher prevalence of low muscle mass in our participants with normal BMI. Small calf circumference was significantly less prevalent in the highest FMI, FM/FMI and FFMI tertile. Sarcopenic obesity was significantly more prevalent in the highest FMI and FM/FMI tertile. Higher fat mass was associated with slower gait speed.

Until recently, BMI has been reported as a useful predictor of metabolic diseases but studies have only shown moderate correlation for diabetes and other chronic diseases (47). BMI does not distinguish between metabolically healthy and metabolically abnormal obesity. A large number of cohort studies have shown that obesity defined by high BMI is not necessarily associated with increased mortality but rather associated with a favorable prognosis especially for survival in heart failure, hypertension, peripheral vascular disease and chronic kidney disease (48). This is termed the “obesity paradox” and most studies used BMI to define obesity which may not differentiate between fat mass and fat free mass (49). A recently published study on BMI and waist circumference showed that high BMI was associated with better functional and cognitive status especially in males (15). Males in our study had significantly higher FFMI and highest tertile of FFMI group had significantly higher MoCA scores. High BMI is a composite of fat mass and fat free mass, and BMI cannot distinguish between fat mass, fat free mass and distribution of adipose tissue. Instead of BMI, body fat percentage, FFMI or FM/FFM ratio may better classify those at higher risk of mortality and functional decline.

There were gender differences where FFMI was significantly higher and FM/FFM significantly lower in male while body fat percentage and FMI being significantly higher in females across all age groups. Similar findings have been shown even in normal population (17). Unlike other studies, there were no significant differences with increasing age within gender possibly explained by similar frailty status (17). The FM/FFM is influenced by age, gender, and BMI. In females, the ratio has been found to increase in middle-aged women, and decline after the age of 70 years (18). In our female participants, the FM/FFM ratio declined after the age of 80 years old. In male, there is a linear relationship with age, and U-shaped curvilinear association in the obese group (18).

Chronic low-grade inflammation with ageing and obesity is associated with diabetes and frailty (50). Almost two thirds of our study participants in the highest FMI, FFMI and FM/FFM tertiles had diabetes. Earlier study has shown that older age, central fat distribution and increase in FFMI especially in female were associated with metabolically abnormal obesity and higher odds of diabetes in obese Asian adults (51). Another recent study by LeCroy et al. showed higher incidence of prediabetes and greater increase in insulin and glucose measures in those with obesity and greatest percentage decrease in the relative fat free mass over 6 years (52). The participants in our study in the highest tertile had a very high mean BMI and 13.6% of the highest FFMI tertile participant experienced significant weight loss in the past 6 months. Weight loss in obese older adults without resistance exercise and protein enhanced diet is known to cause accelerated muscle mass loss (53). Reducing visceral fat mass, and increasing FFM may reduce the risk of diabetes mellitus (54). Comprehensive lifestyle intervention including healthy diet in combination with exercise has shown to significantly increase fat free mass and/or reduce percentage body fat both in the adolescent and older adults (55, 56).

For those in highest the FMI, FM/FFM and lowest FFMI tertile, only 1 in 10 fulfilled the WHO physical activity recommendations. Sedentary lifestyle has been associated with increase in fat mass and weight gain (57). Weight gain and fat mass have been shown to be associated with slow gait speed which can lead to a downward spiral in physical activity (58). In prior studies, risk of losing mobility has been significantly associated with very high (>80th percentile) BMI (59, 60) and in women who experienced >5% of weight loss suggesting greater loss of FFMI (60). Exercise but not diet induced weight loss has shown to reduce muscle inflammation markers and increase anabolism in frail older adults (61). Exercise has been shown to be beneficial in reducing adipose tissue, improving muscle and bone mass, and preventing chronic diseases (53, 56, 62).

Increasingly, phase angle is being considered as an indicator of nutritional status, muscle quality, muscle size and function, cellular integrity, and predictor of mortality (63–67). Phase angle was significantly higher in the lowest FMI and FM/FFM tertile and highest FFMI tertile. Body cell mass is a measure of metabolically active tissue mass, nutrition, and thought to be a more reliable indicator of loss of muscle mass with ageing (68). Body cell mass was significantly higher in the lowest FM/FFM and highest FFMI tertile.

Calf circumference is positively correlated with muscle mass, associated with nutritional status and disability, and recommended by the Asian Working Group for Sarcopenia as a screening tool for sarcopenia (31, 69, 70). Interestingly, our study found prevalence of small calf circumference was significantly lower in higher FMI and FM/FMI tertiles where the prevalence of sarcopenic obesity was also high. Calf circumference has been shown to not be a good predictor of lean muscle mass especially in women with high body fat as it reflects both high muscle and fat content (71). If calf circumference is used as a screening tool for sarcopenia, a large proportion of older adults with high fat mass may be wrongly classified as non-sarcopenic.

There is still an ongoing debate on the diagnosis and the definition of sarcopenia which continues to evolve. Body composition including fat mass and fat free mass plays an important role in diagnosis and management of sarcopenia. Most sarcopenia definitions have included gender specific cut-offs for ASM and applied the same cut-off for sarcopenia across the BMI range from normal to obese without considering the relative contribution of FMI and/or FFMI to physiological function and adipose tissue dysfunction. Muscle quality was only recently added to the definition in 2019 (31). Loss of muscle mass with aging is a complex process, has multiple causes and is interdependent on other aspects of body composition (72). Increasingly, it is recognized that muscle doesn’t function as a single organ but rather through the concept of bone-muscle and fat crosstalk, where the metabolic effects of the three structures are interdependent on each other through autocrine, paracrine and endocrine effect (24). Identifying appropriate definition of sarcopenia is crucial in raising awareness, upstream interventions, resource allocation and preventing disability.

### Strength and Limitations of Study

The strength of our study includes involvement of community dwelling older adults and objective measurements of muscle strength, and body composition. The limitation is the cross-sectional design which did not allow us to evaluate longitudinal changes of physical function with change in FMI, FFMI and FM/FFM. Our study included only pre-frail participants with normal or high BMI and findings cannot be generalized to the entire population. In addition, we did not adjust for nutrition intake. The subgroup sample size is small as this was not the primary aim of the multidomain study. Nonetheless, high FFMI did correlate with better physical and cognitive function in this group. Low muscle mass was more prevalent in the normal BMI group in the pre-frail. We did not investigate the multifactorial causes of sarcopenia. Lastly, body composition measured by BIA may be affected by hydration, edema and fasting.

While higher FFMI and lower FMI/FMI was beneficial and associated with better physical and cognitive function, there is an urgent need to have normative data based on age, ethnicity, gender, and validation carried out for risk prediction of cardiometabolic, sarcopenia and musculo-skeletal disorders (73).

## Conclusion

BMI cannot distinguish between fat mass, fat free mass and fat distribution. FFMI and FM/FFM may be a better measure of cognitive and functional outcome in older adults. In pre-frail older adults, there was a higher prevalence of low muscle mass in the normal BMI group. Higher BMI was associated with lower odds of sarcopenia in the pre-frail. Higher phase angle and body cell mass was more prevalent in the lower FM, FM/FFM and higher FFMI tertile which are indicators of nutritional status, muscle quality, cellular integrity and mortality. The role of calf circumference as a screening tool for sarcopenia in those with high BMI and cut-off values for healthy BMI values needs to be validated in larger population. Health promotion and frailty prevention intervention should focus on FFMI increment.

## Data Availability Statement

The raw data supporting the conclusions of this article will be made available by the authors, without undue reservation.

## Ethics Statement

The studies involving human participants were reviewed and approved by Domain Specific Review Board, National Healthcare Group, Singapore. The patients/participants provided their written informed consent to participate in this study.

## Author Contributions

Data cleaning and analysis: RM and MW. Figure and table design: RM and MW. Writing and methodology: RM. Writing, review and editing: RM, MW, MC, LT, BW, RH, JS, LA, SS, and JM. All authors contributed to the article and approved the submitted version.

## Funding

This study is sub-study of a larger study that has been funded by Ministry of Health of Singapore: Healthy Ageing Innovation Grant under National Innovation Challenge on Active and Confident Ageing (Award No: MOH/NIC/HAIG02/2017) and National Medical Research Council (HSRG-HP17Jun003).

## Conflict of Interest

The authors declare that the research was conducted in the absence of any commercial or financial relationships that could be construed as a potential conflict of interest.

## Publisher’s Note

All claims expressed in this article are solely those of the authors and do not necessarily represent those of their affiliated organizations, or those of the publisher, the editors and the reviewers. Any product that may be evaluated in this article, or claim that may be made by its manufacturer, is not guaranteed or endorsed by the publisher.
